# Mechanisms underlying the antihypertensive properties of *Urtica dioica*

**DOI:** 10.1186/s12967-016-1017-3

**Published:** 2016-09-01

**Authors:** Rahila Qayyum, Hafiz Misbah-ud-Din Qamar, Shamim Khan, Umme Salma, Taous Khan, Abdul Jabbar Shah

**Affiliations:** Department of Pharmacy, COMSATS Institute of Information Technology, University Road, Abbottabad, KPK 22060 Pakistan

**Keywords:** *Urtica dioica*, Antihypertensive, Vasorelaxant, Endothelium-dependent and-independent

## Abstract

**Background:**

*Urtica dioica* has traditionally been used in the management of cardiovascular disorders especially hypertension. The aim of this study was to explore pharmacological base of its use in hypertension.

**Methods:**

Crude methanolic extract of *U. dioica* (Ud.Cr) and its fractions (Ud.EtAc, Ud.nHex, Ud.Chl and Ud.Aq) were tested in vivo on normotensive and hypertensive rats under anesthesia for blood pressure lowering effect. In-vitro experiments on rat and rabbit aortae were employed to probe the vasorelaxation mechanism(s). The responses were measured using pressure and force transducers connected to PowerLab Data Acquisition System.

**Results:**

Ud.Cr and fractions were found more effective antihypertensive in hypertensive rats than normotensive with remarkable potency exhibited by the ethyl acetate fraction. The effect was same in the presence of atropine. In isolated rat aortic rings, Ud.Cr and all its fractions exhibited l-NAME sensitive endothelium-dependent vasodilator effect and also inhibit K^+^ (80 mM)-induced pre-contractions. In isolated rabbit thoracic aortic rings Ud.Cr and its fractions induced relaxation with more potency against K^+^ (80 mM) than phenylephrine (1 µM) like verapamil, showing Ud.EtAc fraction the most potent one. Pre-incubation of aortic rings with Ud.Cr and its fractions exhibited Ca^2+^ channel blocking activity comparable with verapamil by shifting Ca^2+^ concentration response curves to the right. Ud.Cr and its fractions also ablated the intracellular Ca^2+^ release by suppressing PE peak formation in Ca^2+^ free medium. When tested on basal tension, the crude extract and all fractions were devoid of any vasoconstrictor effect.

**Conclusions:**

These data indicate that crude methanolic extract and its fractions possess antihypertensive effect. Identification of NO-mediated vasorelaxation and calcium channel blocking effects explain the antihypertensive potential of *U. dioica* and provide a potential pharmacological base to its medicinal use in the management of hypertension.

## Background

*Urtica dioica* L., (Urticaceae) is one of such plants, used by local practitioners for various human ailments [1]. *Urtica dioica* is commonly known as “Nettle”, which comes from word meaning “textile plant” because of its use in the production of silky fabrics [2, 3]. Locally it is known as “Bichu bute” in Urdu, “Kali and Kandale” in Hindko and “Zhoomi joomi” in Shina. It is found in different areas of Pakistan like Hazara Division, Gilgit Baltistan, Naran, Kaghan and Balochistan. In addition to this, it is widely distributed throughout the USA, Europe and Himalaya naturally, as well as cultivated commercially [4].

*Urtica dioica* is an annual growing to 0.6 m tall shrub which bears opposite, cordate, deeply serrate, pointed leaves which are downy underneath. Flowering and fruiting time is from June to October. The stem and leaves of the plant are covered with stinging trichomes. The fluid present in the trichomes is histamine, 5-hydroxyl tryptamine, acetylcholine, small amount of formic acid and leukotrienes which enter the skin and causes blistering. The plant prefers to grow on loose soil with organic matter rich in nitrogen and high phosphate levels for rapid growth. This plant can be propagated through seeds or vegetative by divisions. It is a relief that nettles can be established from cuttings so there is potential to cultivate both male and female forms [5].

Traditionally *U. dioica* has been used for the treatment of hypertension, gastro intestinal, hepatic disorders [6] and diabetes [7, 8].

The extract of this plant contains different chemical compounds including neophytadiene (25.21 %), sinapic acid (25 %), phthalic acid (8.15 %), dibutyl phthalate (7.37 %), bis(2‐ethyl hexyl) maleate (6.32 %) and 1,2‐benzene di carboxylic acid (7.62 %) [9]. Nettle is nutritionally high in vitamins A, C and D, also minerals iron, manganese, potassium and calcium [5]. Neophytadiene and sinapic acid are reported to be an antibacterial compound [10]. Neophytadiene is suitable for treatment of headache, rheumatism and some skin diseases [11]. Aromatic compounds including carboxylic acids and esters were also reported in this plant [12]. Finally, fat acids including phthalic acid, dibutyl ester, bis(2‐ ethyl hexyl) maleate and 1,2‐benzenedi carboxylic acid were isolated. These compounds are reported to have anti-putrefying [13] and antimicrobial effects [14]. *Urtica dioica* exerts different pharmacological effects, such as a stimulation of human lymphocyte proliferation [15], an anti-inflammatory [16], and used in the treatment of prostatic hyperplasia [17]. There are also reports of diuretic and natriuretic effects of *U. dioica* [18].

The reported diuretic activity and traditional medicinal use of *U. dioica* in hypertension indicates it might have beneficial effects on the cardiovascular system. This investigation was therefore carried out to explore the antihypertensive potential and underlying vascular mechanisms of *U. dioica* using in vivo and in vitro experimental approaches.

## Methods

### Plant material

Dried rhizome of *U. dioica* L., were purchased from a known herbalist (Ali Pansar, Sargodha) in Sargodha, Punjab, Pakistan and identified by botanist Dr. Ghulam Murtuza Shah, Assistant Professor, Post Graduate College, Abbottabad. The sample voucher (Ud.Rh.08/12) was deposited to the Department of Pharmacy, COMSATS Institute of Information Technology, Abbottabad. After cleaning of adulterant material, the rhizomes were ground into coarse powder. Extraction and fractionation was carried out as described previously [19]. About 5 kg of coarse powder was soaked in methanol at room temperature (23–25 °C) for 15 days with occasional shaking. It was filtered through eightfold muslin cloth and then through a Whatman qualitative grade 1 filter paper. This procedure was repeated twice and the combined filtrate was evaporated on rotary evaporator under reduced pressure, yielding approximately 10.81 % crude extract (Ud.Cr).

Fractionation was carried out, using solvents of increasing polarity. Ud.Cr was mixed in distilled water and equal volume of *n*Hexane was added to it and shaken vigorously in a separating funnel. The *n*Hexane, upper layer was collected and evaporated on rotary evaporator to give the *n*Hexane fraction (Ud.nHex: yielding 16 %). The lower layer was taken in a separating funnel, and equal volume of chloroform was added. The chloroform layer (lower) was collected and evaporated on rotary evaporator to obtain the chloroform fraction (Ud.Chl: yielding 24 %). The other layer (upper) was again taken into a separating funnel, ethyl acetate was added into it, separated and was also evaporated in rotary evaporator to give the ethyl acetate fraction (Ud.EtAc: yielding 21 %). The remaining lower layer was collected, evaporated and considered as the aqueous fraction (Ud.Aq: yielding 39 %).

### Chemicals

Acetylcholine chloride (ACh), atropine sulphate, phenylephrine hydrochloride (PE), norepinephrine (NE), potassium chloride, N_ω_-nitro l-arginine methyl ester (l-NAME) hydrochloride and verapamil hydrochloride were purchased from Sigma Chemicals Company, St. Louis, MO, USA. Pentothal sodium and heparin injections were obtained from Abbot Laboratories, Karachi, Pakistan and F. Hoffmann-La Roche, Basel, Switzerland, respectively. Stock solutions of the drugs were made in distilled water/saline and the subsequent dilutions were prepared fresh on the day of experiment.

### Experimental animals and housing conditions

Rabbits (1–1.5 kg) and Sprague–Dawley rats (200–220 g) preferably male and local bred were housed at the Animal House of the COMSATS Institute of Information Technology, Abbottabad, maintained at 23–25 °C. Experiments performed complied with the rulings of the Institute of Laboratory Animal Resources, Commission on Life Sciences, National Research Council [20] and approved by the Ethical Committee of COMSATS Institute of Information Technology, Abbottabad, in its meeting held on 17-06-2013 vide notification EC/PHM/07-2013/CIIT/ATD.

### In-vivo experiments

#### Blood pressure measurement in normotensive anesthetized rats

These experiments were performed on male Sprague- Dawley rats (200–220 g) as described [21]. Animals were anesthetized with an intra-peritoneal injection of sodium thiopental (pentothal, 40–100 mg/kg), fixed in a supine position on a dissecting table; a small mid tracheal incision (approximately 1 cm) was made to expose trachea, right jugular vein, and carotid artery. The trachea was cannulated with a polyethylene tubing PE-20 and cleaned from time to time to maintain the spontaneous respiration. The right jugular vein was cannulated with a polyethylene tubing PE-50 to facilitate the intravenous infusions of the standard drugs and test materials. The carotid artery was cannulated with similar tubing filled with heparinized saline (60 IU/mL) and connected to a pressure transducer coupled with PowerLab (ML 846) Data Acquisition System (ADInstruments Australia). This connection was used for blood pressure recording. The exposed surface was covered with a piece of tissue paper moistened with warm saline. Rats were infused with heparinized saline (0.1 mL) to prevent blood clotting. The body temperature of the animal was maintained by using an overhead lamp.

#### Experimental protocol

After 20–30 min of the equilibrium period, acetylcholine and norepinephrine were used to check the stability of the animals toward hypotensive and hypertensive responses, respectively. Acetylcholine (1 µg/kg) in a volume of 0.1 mL was slowly injected followed by a flush of 0.1 mL saline, which caused a fall in blood pressure. After approximately 5–10 min later, when the normal pattern of blood pressure was resumed, norepinephrine (1 µg/kg) was slowly injected followed by a flush of 0.1 mL saline, which caused an increase in blood pressure. After resuming the normal pattern of blood pressure, rats were then injected intravenously with 0.1 mL saline or with the same volume of test substances. The mean arterial pressure (MAP) was allowed to return to the resting level between injections. Drugs, extract, and their fractions were then injected intravenously and followed by a flush with 0.1 mL saline. Changes in MAP were recognized as the difference between the steady-state values before and the lowest readings after injection. The MAP was calculated as the diastolic blood pressure (BP) plus one-third pulse width (systolic BP–diastolic BP).

#### Blood pressure measurement in hypertensive anesthetized rats

The protocol of Lawler et al. and Vasdev et al. [22, 23] was followed with some modifications. Sprague–Dawley male rats (200–220 g; n = 5) were hygienically housed in uniform conditions. The rats were given high-salt (8 % NaCl) diet and water ad libitum for 6-weeks. One day prior to the experiment, the rats were given normal diet and water. Subsequently the rats were used for in vivo blood pressure measurement as described earlier.

### In-vitro experiments

#### Rat thoracic aorta

The procedure of Taqvi et al. [24] was followed with some modifications. Thoracic aorta was isolated from Sprague–Dawley rats of normotensive and high salt-induced hypertensive rats carefully to avoid any damage to the endothelium. The aorta was then transferred into the Kreb’s solution aerated with carbogen (5 % CO_2_ in O_2_). The composition of Kreb’s solution was (mM): NaCl 118.2, NaHCO_3_ 25.0, CaCl_2_ 2.5, KCl 4.7, KH_2_PO_4_ 1.3, MgSO_4_ 1.2 and glucose 11.7 (pH 7.4). It was cautiously cleaned off fats and other connective tissues and then cut into rings 2–3 mm wide. In some rings, the endothelium was intentionally removed by gentle rubbing of the intimal surface with forceps. The rings with intact endothelium that produced less than 80 % relaxation in response to acetylcholine (1 µM) were tossed away. Individual rings were suspended in 10 mL tissues baths at 37 °C aerated with carbogen. A preload of 1 g was applied to each preparation and incubated for 30 min. Changes in isometric tension were recorded and analyzed through a force transducer (MLT 0201) coupled with a bridge amplifier (N12128) and PowerLab (ML 846) Data Acquisition System (ADInstruments).

#### Endothelium-dependent and-independent effects

A series of experiments were conducted to assess endothelium-dependent or independent effects of Ud.Cr and its fractions on isolated aortic rings of normotensive and hypertensive rats. When the tension was at resting state or reached a plateau induced by PE (1 µM), Ud.Cr and its fractions (mg/mL) were cumulatively added into the organ bath. The rings with intact and denuded endothelium were always tested in parallel. To determine the underlying mechanisms, endothelium-intact rings were incubated with l-NAME (10 µM), for 30 min before the addition of PE. The test material was then added cumulatively and the concentration response curves (CRCs) were constructed for the inhibitory responses.

K^+^ (80 mM) was also used to depolarize the tissue, which produced sustained contractions and allowed to study the effect on the VDCs. The test material was then added cumulatively and relaxation was expressed as the percent of the contractions induced by K^+^ (80 mM). This protocol was used to see if there is difference in vascular reactivity in two different vascular preparations from different species [25].

#### Rabbit thoracic aorta

As described previously [22, 25] rabbits were killed by a blow on the back of the head; the thoracic aorta was removed and cut into rings of approximately 2–3 mm width. Aortic rings were suspended between a pair of stainless steel hooks in 10 mL organ baths, one hook was anchored to a steel rod at the bottom and the other was attached to a force transducer (MLT 0201). The tissues were suspended in normal Kreb’s solution, maintained at 37 °C, and continuously bubbled with 5 % CO_2_ in O_2_ (carbogen). A resting tension of 2 g was applied to each tissue and an equilibrium period of 1 h was allowed before studying the effect of test materials. Phenylephrine (1 µM) was used to stabilize the preparations. Changes in isometric tension were recorded and analyzed through a force transducer coupled with a bridge amplifier and PowerLab Data Acquisition System (ADInstruments, Sydney, Australia).

#### Effect on contraction induced by phenylephrine and K^+^ (80 mM)

The protocol of Chan et al. [26] was followed with some modifications. Phenylephrine (1 µM) or high K^+^ (80 mM) was used to induce steady-state contractions. The plant extract and its fractions were added cumulatively to obtain concentration response relationship and the relaxation was expressed as percent of agonist-induced contractions. Vascular reactivity of the extract was evaluated on Ca^2+^ influx either through voltage-dependent (VDCs) or receptor-operated Ca^2+^ channels (ROCs) and Ca^2+^ release from internal store(s).

#### Determination of calcium channel blocking activity

In the first set of experiments, an attempt was made to see if the relaxation induced by the extract involved Ca^2+^ influx through VDCs. Aortic rings were washed four to five times with Ca^2+^-free solution before the construction of control CRCs of Ca^2+^ (as CaCl_2_). When the control CRCs of Ca^2+^ were found superimposable (usually after two cycles), then tissue was pretreated with the plant extract for 30–45 min to test the possible calcium channel blocking effect. A parallel control was also run under similar experimental conditions. A second set of experiments was used to elucidate whether Ud.Cr and its fractions induce relaxation through ROCs. Aortic rings were washed four to five times with normal Kreb’s solution and the effect of Ud.Cr and its fractions was determined on PE (1 µM)-induced sustained contractions [22].

#### Effect on intracellular Ca ^2+^ stores

In a set of experiments, the aim was to clarify whether the relaxation induced by Ud.Cr and its fractions is related to inhibition of intracellular Ca^2+^. The rings were exposed to Ca^2+^-free solution for 15 min before the application of PE (1 µM) to induce the first transient contraction. The composition of Ca^2+^-free/EGTA Kreb’s solution was (mM): NaCl 118.2, NaHCO_3_ 25.0, KCl 4.7, KH_2_PO_4_ 1.3, MgSO_4_ 1.2, EGTA 0.05, and glucose 11.7 (pH 7.4). The rings were then washed three times with normal Kreb’s solution and incubated for at least 40 min for refilling of the intracellular stores. Subsequently, the medium was rapidly replaced with Ca^2+^-free solution and the rings were incubated for another 15 min. The second contraction was then induced by PE (1 µM) in the presence of Ud.Cr and its fractions (mg/mL), which were added 30 min before the application of PE, both contractions were compared [22].

#### Statistical analysis

Data obtained from the animal and in vitro experiments were expressed as the mean ± standard error (±SEM). Statistical difference between the treatments and the control were evaluated by a one-way analysis of variance(ANOVA) followed by Tukey’s multiple comparison test using IBM SPSS software (Version 20, SPSS Inc., Chicago, IL). Differences were considered significant at *p < 0.05, **p < 0.01, and ***p < 0.001.

## Results

### Effect on blood pressure in normotensive anesthetized rats

**Fig. 1:**
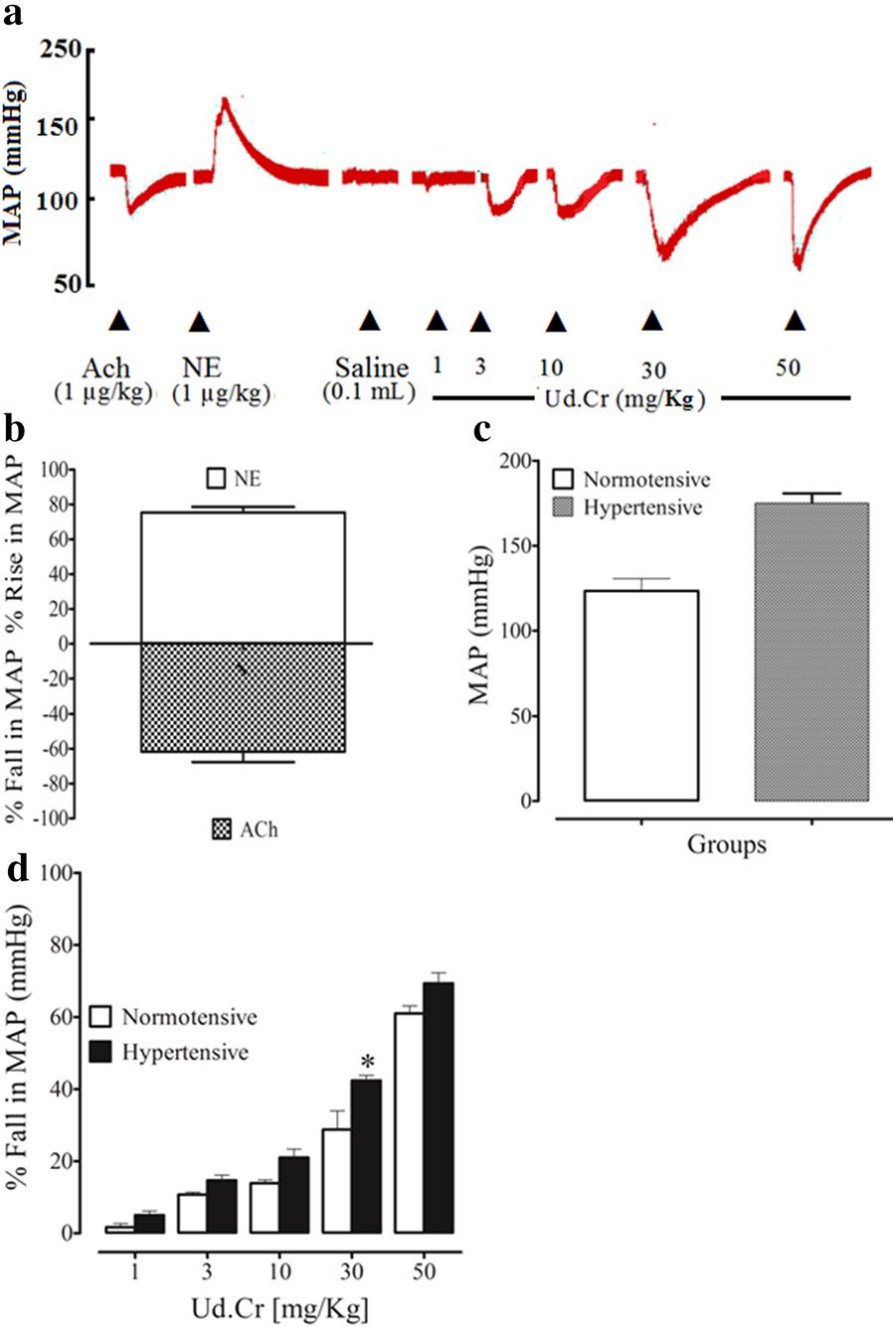
A representative tracing **a** showing the effect of norepinephrine (NE), acetylcholine (Ach), and different doses of crude extract of *U. dioica* (Ud.Cr) on mean arterial pressure (MAP) in normotensive rats under anesthesia. **b** The hypertensive and hypotensive effects of norepinephrine (NE) and acetylcholine (ACh), respectively. **c** The blood pressure of normotensive and hypertensive rats. **d** The effect of crude extract of *U. dioica* (Ud.Cr) on MAP in normotensive and hypertensive rats, under anesthesia (n = 6–7), *p < 0.05, **p < 0.01, and ***p < 0.001 represent the significance difference between the % fall in MAP on normotensive and hypertensive rats

**Fig. 2:**
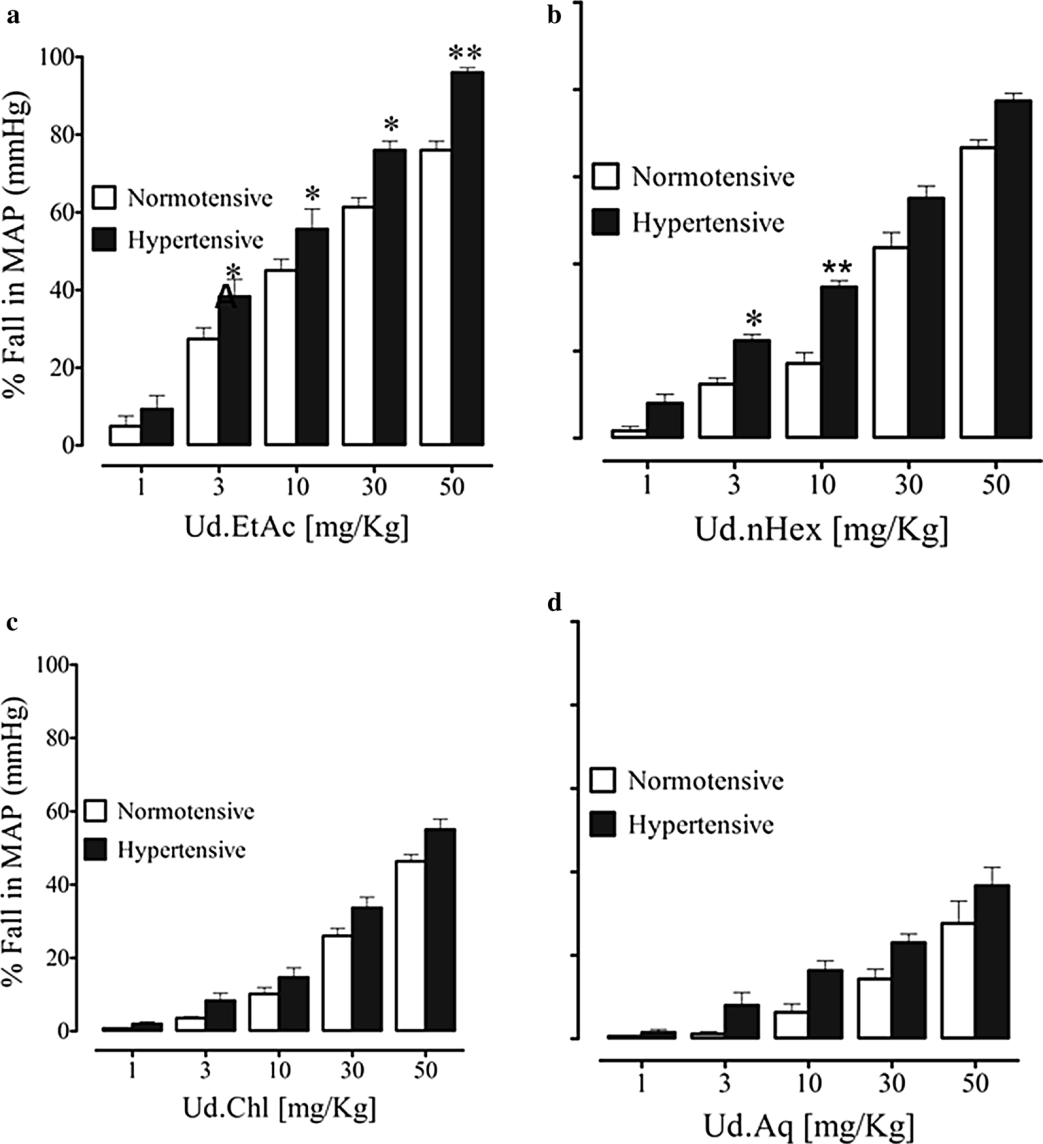
*Graphs* show the effect of **a** ethylacetate (Ud.EtAc) **b** *n*Hexane (Ud.nHex), **c** chloroform (Ud.Chl) and **d** aqueous (Ud.Aq) fractions of *U. dioica* on mean arterial pressure (MAP) in normotensive and hypertensive rats, under anesthesia. Values shown are mean ± SEM (n = 6–7) *p < 0.05, **p < 0.01, and ***p < 0.001 represent the significance difference between the % fall in MAP on normotensive and hypertensive rats

Before the administration of the crude extract and fractions of *U. dioica* L., standard drugs such as acetylcholine and norepinephrine were used, they caused a fall and rise in MAP, respectively (Fig. 1a). The MAP in normotensive and high salt induced hypertensive rats was 115 ± 5.12 (n = 20) and 166 ± 4.45 (n = 20), respectively. In normotensive rats under anesthesia, intravenous administration of Ud.Cr caused a fall in MAP (Fig. 1a). The % fall in MAP at respective doses of 1, 3, 10, 30 and 50 mg/kg was 1.68 ± 2.3, 10.74 ± 1.8, 13.85, 7 ± 2.7, 28.81 ± 3.7 and 61.00 ± 8.06 (Fig. 1d). Among fractions tested, all fractions caused a fall in MAP, but the ethyl acetate fraction being the most potent at 50 mg/kg (76.00 ± 3.77) (Fig. 2). The percent fall in each case was statistically different (p < 0.05) compared to pre-treated values among the different doses. To check the possibility of involvement of muscarinic receptors, rats were pre-treated with atropine (1 mg/kg). This pretreatment did not affect the blood pressure lowering effect of the extract and fractions (data not shown).

### Effect on blood pressure in hypertensive anesthetized rats

In hypertensive rats under anesthesia, intravenous administration of Ud.Cr caused a fall in MAP more than the normotensive rats. The % fall in MAP at respective doses of 1, 3, 10, 30 and 50 mg/kg was 5.00 ± 3.4, 14.66 ± 1.5, 21.00 ± 0.27, 42.33 ± 0.95 and 69.33 ± 2.51 (Fig. 2). All fractions have blood pressure lowering effect, but the ethyl acetate fraction was the most potent (p < 0.05), which caused a fall in MAP 9.33 ± 1.9, 38.33 ± 1.25, 55.00 ± 1.7, 76.00 ± 4.1 and 96.00 ± 3.77 (Fig. 2). Pretreatment of the rats with atropine (1 mg/kg) did not affect blood pressure lowering effect of the fractions (data not shown) (Fig. 2).

### Endothelium-dependent and-independent effects

**Fig. 3:**
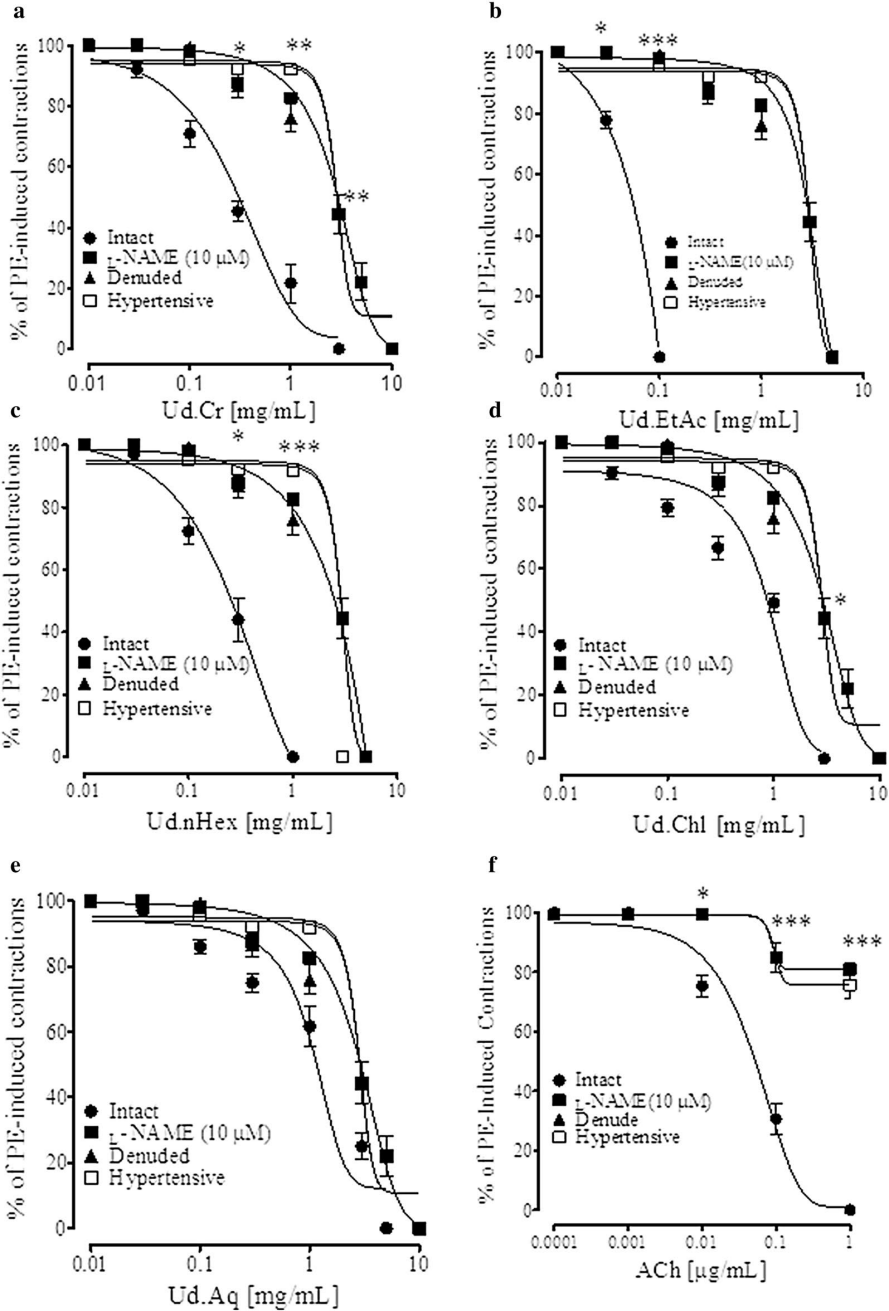
**a**–**f** The response of crude extract of *U. dioica* (Ud.Cr), its fractions ethyl acetate (Ud.EtAc), *n*Hexane (Ud.nHex), chloroform (Ud.Chl) and aqueous (Ud.Aq) and acetylcholine on PE-induced contractions in intact (with and without l-NAME (10 µM) pretreatment), denuded aortic rings from normotensive rats and rings from hypertensive rats. Values shown are mean ± SEM (n = 6–7), *p < 0.05, **p < 0.01, and ***p < 0.001

In rat aortic rings acetylcholine failed to induce less than 80 % relaxation were excluded. In aortic rings with intact endothelium pre-contracted with PE (1 µM), cumulative addition of Ud.Cr caused endothelium-dependent relaxation with EC_50_ value of 0.28 mg/mL (0.11–0.37). Pretreatment of intact aortic rings with l-NAME (10 µM) inhibited the vasorelaxant effect of Ud.Cr with EC_50_ value of 2.91 mg/mL (1.25–3.0) and shifted the CRCs to the right (Fig. 3a).

In denuded endothelium pre-contracted with PE (1 µM), Ud.Cr relaxed the tissue at higher concentrations with EC_50_ value of 2.91 mg/mL (1.02–3.0) and shifted the CRCs to the right as compared to intact endothelium. In aorta rings of hypertensive rats pre-contracted with PE, Ud.Cr relaxed the tissue at higher concentrations with overlapping EC_50_ value of 2.91 mg/mL (1.02–3.01) observed in the presence of l-NAME or denuded aorta (Fig. 3a).

Intact rat aorta rings pre-contracted with PE (1 µM), cumulative addition of Ud.*n*Hexane induced endothelium-dependent vasorelaxation. This relaxation was inhibited in intact aortic rings pre-treated with l-NAME (10 µM), aortic rings without endothelium and in rings of hypertensive rats. Chloroform and aqueous fractions also induced endothelium-dependent vasorelaxation, similar to the parent crude extract. Interestingly, the effect of ethyl acetate fraction was about 60 times more potent in the aortic rings with intact endothelium, compared to intact rings pretreated with l-NAME and in denuded aortic rings (Fig. 3).

**Fig. 4:**
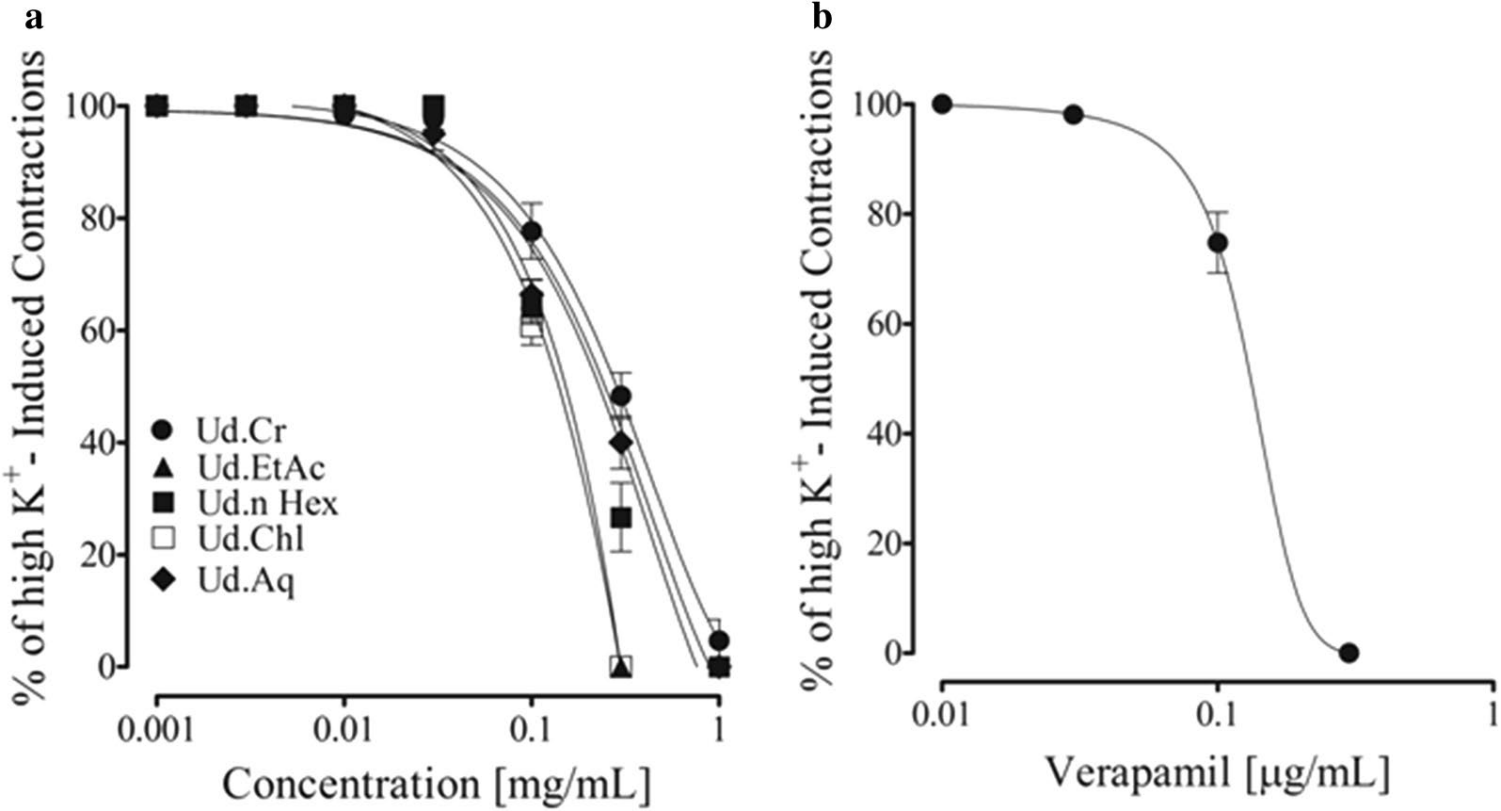
*Graph* shows **a** vasodilator effect of crude extract of *U. dioica* (Ud.Cr), its fractions ethyl acetate (Ud.EtAc), *n*Hexane (Ud.nHex), chloroform (Ud.Chl) and aqueous (Ud.Aq) Ud.nHex, **b** verapamil on high K^+^ (80 mM)-induced contractions in isolated rat aorta rings. Values shown are mean ± SEM (n = 6–7), *p < 0.05, **p < 0.01, and ***p < 0.001 represent the significance difference between the relaxation of intact and denuded aorta

To see effect on vascular smooth muscles, high K^+^ (80 mM) was used. Cumulative addition of the extract and fractions induced relaxation with varying potencies (Fig. 4a). The ethyl acetate fraction being more potent while the aqueous fraction was least, which induced partial inhibition (Fig. 4). Verapamil, a typical calcium channel blocker induced endothelium-independent vasodilator effect (data not shown) with more potency against high K^+^ precontractions (Fig. 4b), as expected.

### Effect on calcium channels

**Fig. 5:**
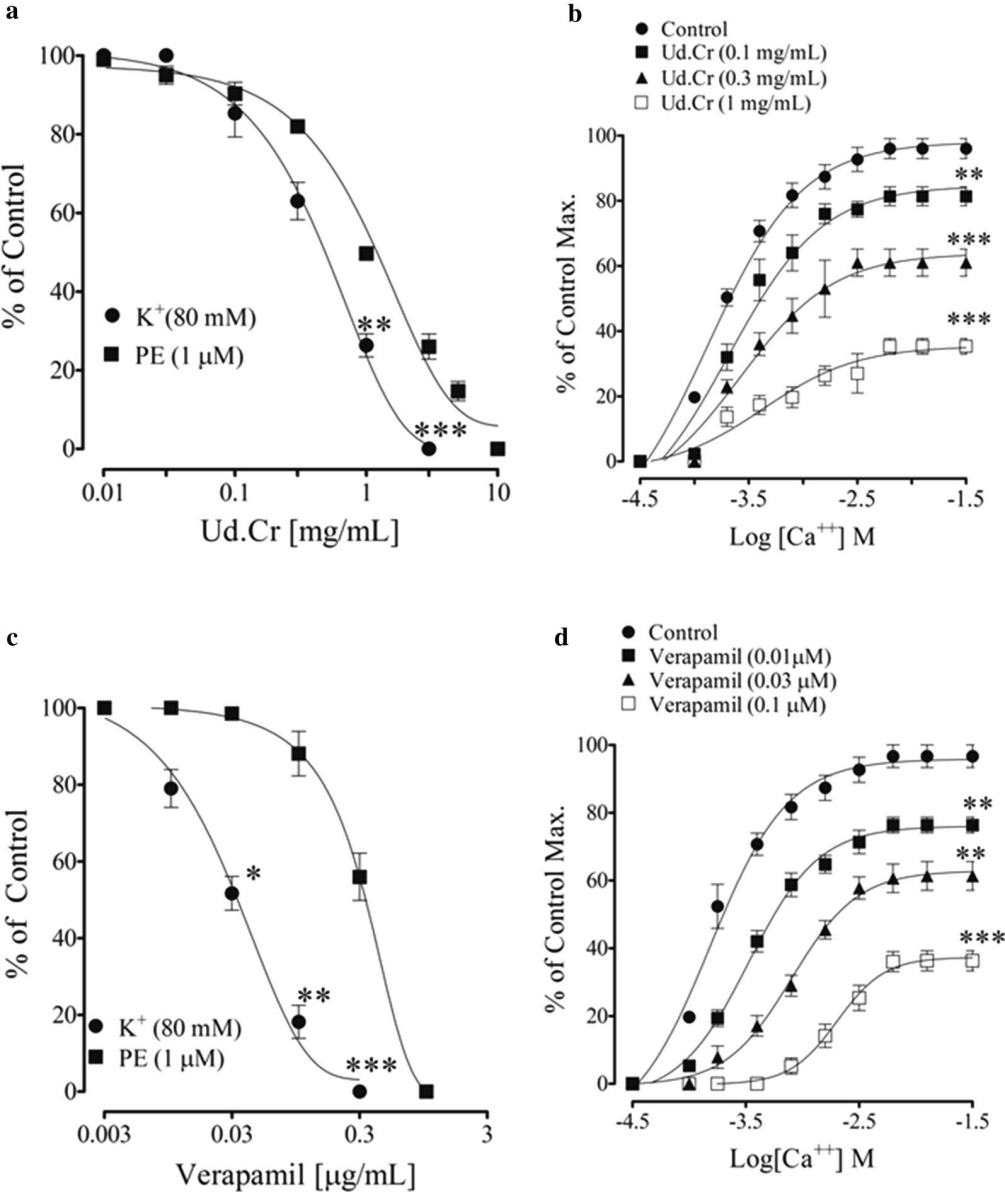
The* graphs* shows the concentration-dependent vasodilator effect of **a** crude extract of *U. dioica* (Ud.Cr), **c** the Verapamil on phenylephrine (PE) and high K^+^(80 mM) precontractions, **b**–**d** respectively, their effect on the Ca^2+^ concentration–response curves, constructed in Ca^2+^- free medium, in isolated rabbit aorta preparations. Values shown are mean ± SEM (n = 6–7), *p < 0.05, **p < 0.01, and ***p < 0.001, represent the significance difference between the relaxation induced by phenylephrine (PE) and high K^+^ (80 mM)

Rabbit aortic rings were used to see effect of the extract and fractions on Ca^2+^ movements through VDCs and store-operated Ca^2+^ channels. Rabbit aortic rings pre-contracted with PE and high K^+^, extract was added cumulatively, which induced a vasodilator effect with more potency against high K^+^ than PE with EC_50_ values of 0.49 mg/mL (0.31–1.75) and 2.19 mg/mL (1.07–3.77), respectively, similar to verapamil (Fig. 5a, c). Pre-incubation of the aortic rings with Ud.Cr (0.1–1.0 mg/mL) shifted the Ca^2+^ CRCs to the right (Fig. 5b), constructed in Ca^2+^-free medium, similar to that caused by verapamil (Fig. 5d).

**Fig. 6:**
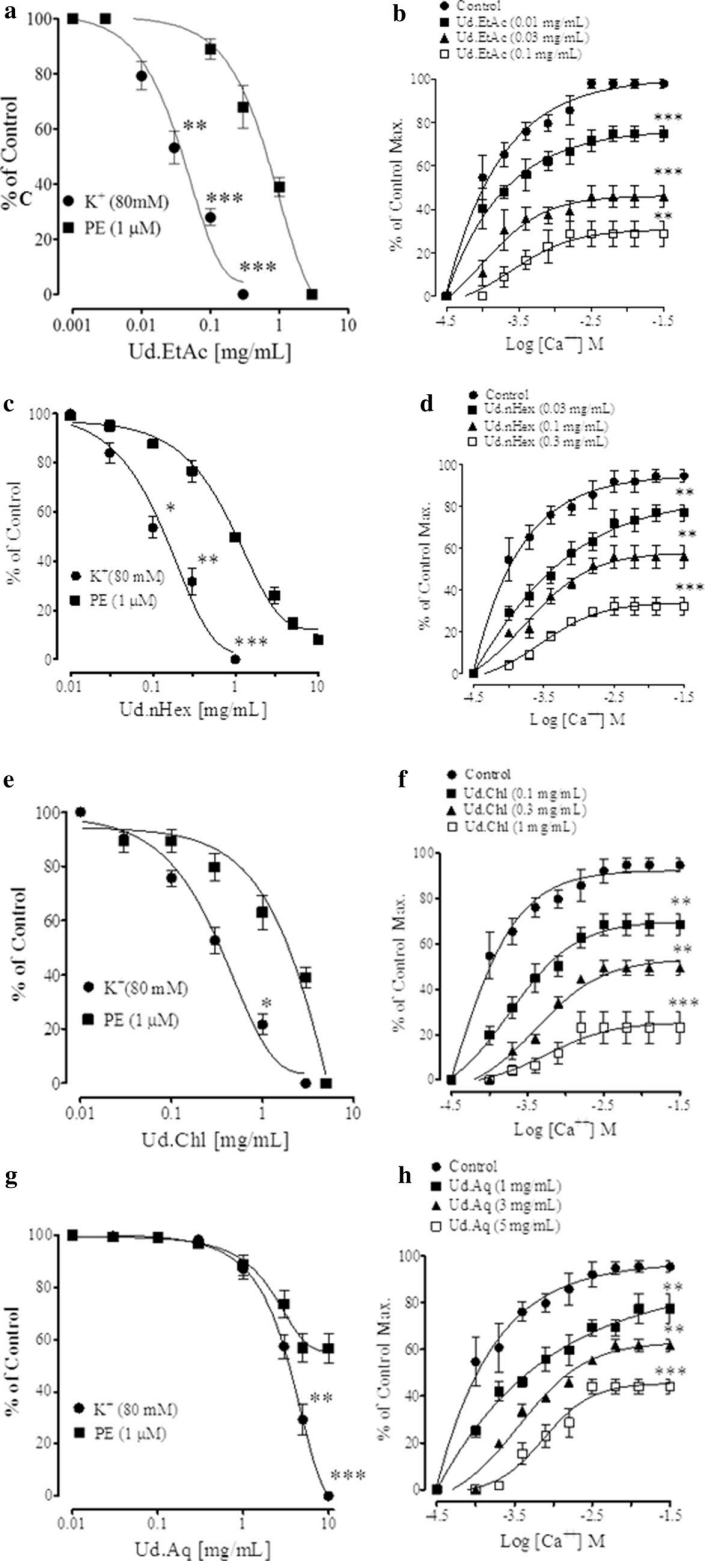
The* graphs* shows the concentration-dependent vasodilator effect of **a**–**g** crude extract of *U. dioica* (Ud.Cr), the Verapamil, ethyl acetate (Ud.EtAc), *n*Hexane (Ud.nHex), chloroform (Ud.Chl), and aqueous (Ud.Aq) fractions on phenylephrine (PE) and high K^+^ (80 mM) precontractions, and (**b**–**h**) respectively, their effect on the Ca^2+^ concentration–response curves, constructed in Ca^2+^- free medium, in isolated rabbit aorta preparations. Values shown are mean ± SEM (n = 6–7), *p < 0.05, **p < 0.01, and ***p < 0.001, represent the significance difference between the relaxation induced by phenylephrine (PE) and high K^+^ (80 mM)

Ethyl acetate fraction was more potent than the parent extract, it induced relaxation of high K^+^and PE pre-contractions with EC_50_ values of 5.14 (3.98–6.31) and 2.02 mg/mL (1.58–2.51), respectively (Fig. 6a). The *n*Hexane fraction was similar to the parent crude extract in potency. However, the chloroform fraction was more and the aqueous fraction was least potent (Fig. 6).

### Effect on calcium stores

**Fig. 7:**
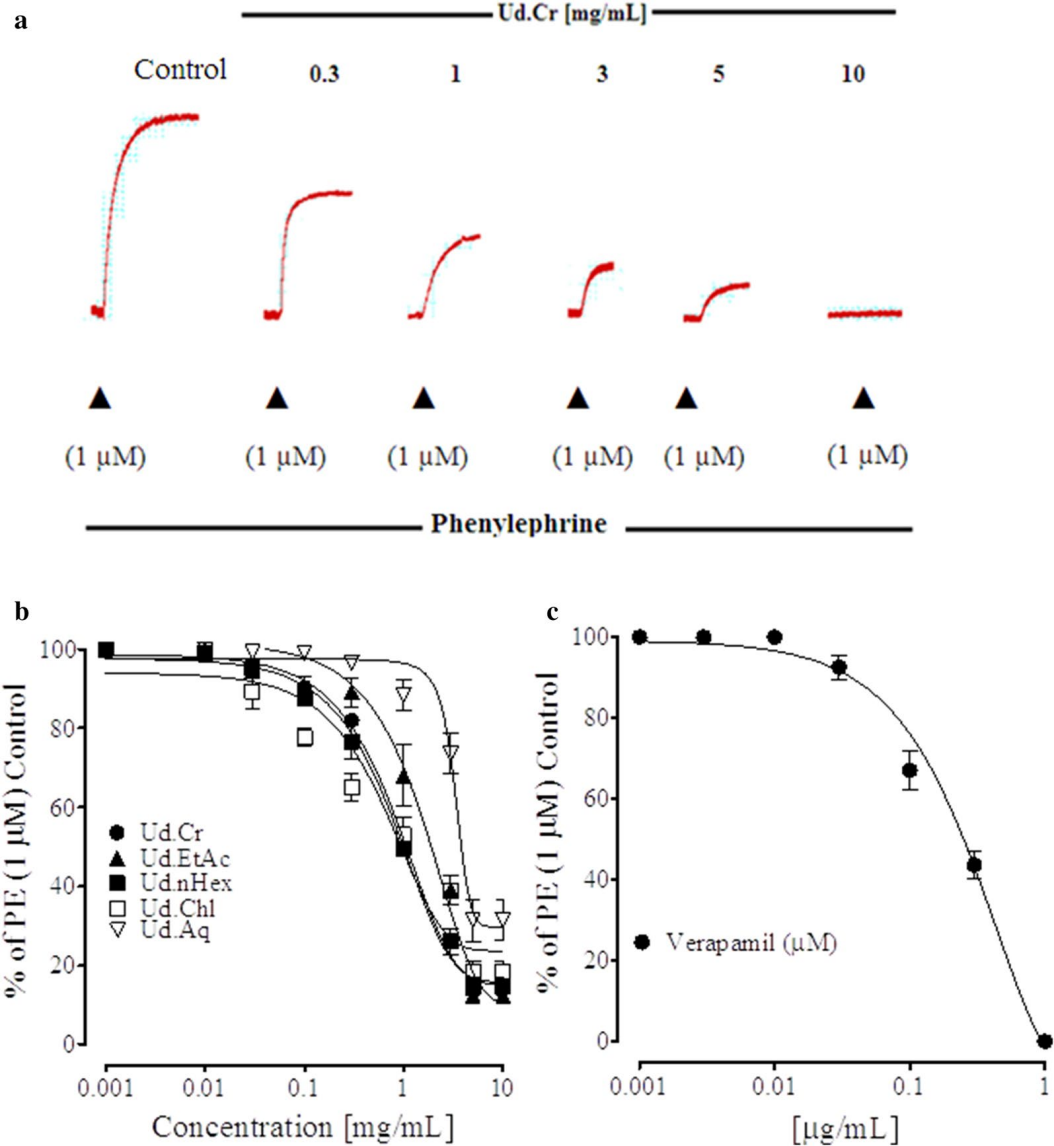
Typical tracing showing inhibitory effect of **a** increasing concentrations of the crude extract of *U. dioica* (Ud.Cr) on the initial peak formation of phenylephrine in Ca^2+^-free medium. **b** The combined effect of Ud.Cr, its ethyl acetate (Ud.EtAc), *n*Hexane (Ud.nHex), chloroform (Ud.Chl), and aqueous (Ud.Aq) fractions, and **c** the effect of verapamil on initial peak formation of PE in isolated rabbit aorta preparations, in Ca^2+^-free medium. Values shown are mean ± SEM (6–7)

In a series of experiments designed to show the effect of Ud.Cr and its fractions on the transient contractile response induced by PE (1 µM), pretreatment of the tissues with Ud.Cr (0.01–10.0 mg/mL) suppressed the PE peak formation in Ca^2+^ –free medium (Fig. 7), similar to that caused by verapamil. The ethyl acetate, *n*Hexane, chloroform, and aqueous fractions (0.01–10.0 mg/mL) also suppressed the transient contractile response of PE (Fig. 7).

## Discussion

For thousands of years, medicine and natural products have been closely interconnected through the use of traditional medicine. Despite competition from other drug discovery methods, natural products are still providing their fair share of new clinical candidates [25]. *Urtica dioica* is traditionally used for the treatment of hypertension [4].

The aim of this study was to confirm the medicinal importance of *U. dioica* in hypertension. Invasive blood pressure monitoring technique was used. This protocol allowed us to observe the effect of extract and fractions directly injected into the systemic circulation. Intravenous injection of the methanolic extract of *U. dioica* and its fractions caused a dose-dependent fall in MAP in both normotensive and hypertensive rats. However the effect was more significant in the hypertensive rats. When compared to the parent crude extract, ethylacetate fraction was more potent, and aqueous fraction was least potent. To see if the blood pressure lowering effect of the extract and fraction might be mediated through muscarinic receptors stimulation, rats were pretreated with atropine, a muscarinic receptor antagonist [27]. This pretreatment did not affect antihypertensive effect either of the extract or fractions. This finding excluded the possibility of muscarinic receptors involvement in the antihypertensive effect of extract or fractions. Blood pressure is the product of peripheral resistance and cardiac output [28]. Therefore, further in vitro experiments were carried out in isolated vascular preparations to determine the underlying mechanisms of antihypertensive effect.

In isolated rat thoracic aorta, Ud.Cr and its fractions induced vasorelaxation on phenylephrin pre-contractions. To see the involvement of mediators of endothelium origin, such as NO and prostaglandins, the aortic rings were pretreated with l-NAME, NO synthase inhibitor [29] and indomethacin, a prostaglandin inhibitor [30]. Aortic ring pretreated with l-NAME ablated the vasorelaxation induced by the crude extract; however indomethacin pretreatment did not affect this relaxation. This indicates that NO is involved in the vasorelaxation induced by the crude extract and prostaglandin, such as PGI2 do not play a role. In aortic rings from hypertensive rats, acetylcholine was failed to induce relaxation, indicating endothelium was damaged with high salt treatment. Interestingly, the relaxation induced by the extract was ablated in denuded rings from normotensive rats and in rings from hypertensive rats, indicates that NO is the major endothelial-derived factor involved in the vasorelaxation. We tested parallel to see if there is any exciting activity in the fractions. We found that the *n*hexane fraction was similar to the parent crude extract. However, the chloroform fraction exhibited a mild endothelium-dependent vasodilator effect while the aqueous fraction induced an endothelium-independent effect. The ethylacetate fraction was exciting, it induced a strong endothelium-dependent NO mediated vasodilator effect with about 60 times more potency than in the intact rings pretreated with l-NAME or denuded rings. This finding indicates that ethylacetate fraction is the most active among the fractions and explains its antihypertensive potency.

Failure of the relaxation induced by the crude extract and fractions in the presence of l-NAME, or in denuded rings or in rings from hypertensive rats, indicates presence of constituents act through different vascular mechanism(s). We tested this hypothesis in rat aortic rings pre-contracted with high K^+^. High K^+^ (80 mM) is known to cause vascular smooth muscle contractions through the opening of voltage dependent L-type Ca^2+^ channels [31], and substance inhibits high K^+^-induced contraction is considered as inhibitor of Ca^2+^ influx [32]. When tested in high K^+^ (80 mM) precontractions, the crude extract and its fractions caused an inhibitory effect, may reflect the restricted Ca^2+^ entry via VDCs and can possibly explain the endothelium-independent component of the vasodilator effect of the extract and fractions. We observed that the effect of high K^+^ (80 mM) precontraction was either weak or not reproducible and sustainable. There is sufficient evidence of heterogenicity of calcium channels [33], they are different in myocardium of rat and rabbit [34] and brain of rat, frog and chicken [35]. Therefore, for further studies we used rabbit aorta in which the effect of high K^+^ is strong and reproducible. Crude and fractions (except the aqueous fraction) induced relaxation of the high K^+^ and phenylephrine precontractions with more potency against high K^+^ than PE, similar to verapamil. Aqueous fraction partially relaxed PE precontractions. The ethylacetate fraction was remarkably more potent (about 49 times) against high K^+^ than PE. This suggests role of the crude extract and fraction inhibiting Ca^2+^ moment though VDCs. This hypothesis was further confirmed when aortic rings pretreated with extract and fraction caused a rightward shift in the CaCl_2_ concentration curves, similar to verapamil.

The relaxation of PE (1 µM) pre-contractions by Ud.Cr and its fractions also reflects its inhibitory effects on Ca^2+^ movement through receptor operated calcium channels (ROCs) and or its release from internal stores. To see possible effect on Ca^2+^ release from the internal store, aortic rings were pretreated with extract and fractions in Ca^2+^ free medium. Interestingly, this pretreatment suppressed PE individual contractions, indicating inhibitory effect on Ca^2+^ release from the internal Ca^2+^ store. Further work is required to explore the underlying mechanism of Ca^2+^ release. These data indicate that the extract of *U. dioica* contains antihypertensive and vasodilator constituents act on both vascular endothelial and smooth muscle cells and release NO and block Ca^2+^ moments. These two mechanisms support the vasodilator effect of the extract and fractions and explain the underlying antihypertensive activity.

We could not identify any particular constituent(s) in the extract responsible for the antihypertensive effect. However, our preliminary phytochemical analysis indicated presence of alkaloids, tannins, flavonoids, saponins, reducing sugars, cardiac glycosides, steroids and terpenoids in the methanolic crude extract of *U. dioica*. Previous studies show that plant derived alkaloids have vasorelaxant properties through multiple pathways like inhibition of calcium release from Ca^2+^ stores [28] and NO pathways [36]. Glucosides [37] and sesquiterpenes [38] also have an inhibitory effect on the cardiovascular system. We assume that these constituents might be the active constituents responsible for the antihypertensive effect of *U. dioica*.

## Conclusion

The current findings on the cardiovascular activities of *U. dioica* and its fractions indicated presence of antihypertensive activity. This antihypertensive effect is the outcome of vasodilation induced by the extract and fractions. NO release and dual inhibitory effect on Ca^2+^ moments through VDCs and release from internal store explains the underlying mechanisms of vasodilation. This finding justifies the medicinal application of *U. dioica* in the management of hypertension, however further studies are required to isolate particular antihypertensive constituent and to underlying molecular mechanisms.

## Abbreviations

Ud.Cr: crude extract of *Urtica dioica*
Ud.nHex: *n*Hexane fraction of *Urtica dioica*
Ud.Chl: chloroform fraction of *Urtica dioica*
Ud.EtAc: ethyl acetate fraction of *Urtica dioica*
Ud Aq: aqueous fraction of *Urtica dioica*
BP: blood pressure
MAP: mean arterial pressure
PE: phenylephrine
Ach: acetylcholine chloride
NE: norepinephrine
l-NAME: N_ω_-nitro l-arginine methyl ester
NO: nitric oxide
Ca^2+^: calcium ion
